# Exhaled Air Dispersion during Coughing with and without Wearing a Surgical or N95 Mask

**DOI:** 10.1371/journal.pone.0050845

**Published:** 2012-12-05

**Authors:** David S. Hui, Benny K. Chow, Leo Chu, Susanna S. Ng, Nelson Lee, Tony Gin, Matthew T. V. Chan

**Affiliations:** 1 Department of Medicine and Therapeutics, The Chinese University of Hong Kong, Hong Kong SAR, The People's Republic of China; 2 Stanley Ho Center for Emerging Infectious Diseases, The Chinese University of Hong Kong, Hong Kong SAR, The People's Republic of China; 3 Center for Housing Innovations, The Chinese University of Hong Kong, Hong Kong SAR, The People's Republic of China; 4 Department of Anaesthesia and Intensive Care, The Chinese University of Hong Kong, Hong Kong SAR, The People's Republic of China; University of North Carolina School of Medicine, United States of America

## Abstract

**Objectives:**

We compared the expelled air dispersion distances during coughing from a human patient simulator (HPS) lying at 45° with and without wearing a surgical mask or N95 mask in a negative pressure isolation room.

**Methods:**

Airflow was marked with intrapulmonary smoke. Coughing bouts were generated by short bursts of oxygen flow at 650, 320, and 220L/min to simulate normal, mild and poor coughing efforts, respectively. The coughing jet was revealed by laser light-sheet and images were captured by high definition video. Smoke concentration in the plume was estimated from the light scattered by smoke particles. Significant exposure was arbitrarily defined where there was ≥ 20% of normalized smoke concentration.

**Results:**

During normal cough, expelled air dispersion distances were 68, 30 and 15 cm along the median sagittal plane when the HPS wore no mask, a surgical mask and a N95 mask, respectively. In moderate lung injury, the corresponding air dispersion distances for mild coughing efforts were reduced to 55, 27 and 14 cm, respectively, *p* < 0.001. The distances were reduced to 30, 24 and 12 cm, respectively during poor coughing effort as in severe lung injury. Lateral dispersion distances during normal cough were 0, 28 and 15 cm when the HPS wore no mask, a surgical mask and a N95 mask, respectively.

**Conclusions:**

Normal cough produced a turbulent jet about 0.7 m towards the end of the bed from the recumbent subject. N95 mask was more effective than surgical mask in preventing expelled air leakage during coughing but there was still significant sideway leakage.

## Introduction

Respiratory tract infections such as influenza and pneumonia predominantly spread by respiratory droplet transmission during coughing whereas contact with fomite is another route of transmission. Several infections are well known to be transmitted by airborne route and these include tuberculosis (TB), varicella and measles [1], [2]. In recent years, there is growing evidence that viral infections such as seasonal influenza may potentially spread by airborne route,[3]–[5], [6] whereas non-invasive ventilation in the presence of an imbalanced medical ward airflow may lead to opportunistic airborne influenza transmission [7].

Cough is a major symptom of respiratory infections such as influenza and severe acute respiratory syndrome[8]–[10]. When an infected person coughs in the upright position, respiratory droplets are released and the maximum air speed may reach 8 m/s [11]. When working in direct contact with patients hospitalized with influenza, standard and droplet precautions are important infection control measures for preventing transmission of influenza in most healthcare situations, in addition to vaccination of healthcare staff, carers and vulnerable patients against seasonal influenza strains [12]. For implementation and facilitation of source control, respiratory hygiene and cough etiquette are important measures to help contain respiratory secretions in persons with respiratory symptoms. In addition, it has been recommended that whenever available, patients who are showing signs of an influenza-like illness should wear a facemask in waiting areas and when they are being transported within the facility [12]. There are however limited data on the aerodynamics of coughing with and without coverage by standard facemasks in the clinical setting.

To advance our knowledge on the infection control measures when managing patients with respiratory infectious diseases, we performed a study to reveal the expelled air during coughing bouts, based on our established laser visualization technique using smoke as a marker, in a high-fidelity human patient simulator (HPS)[13]–[19]. We compared the distance and direction of expelled air during coughing with and without coverage by a surgical mask and a N95 mask in a hospital isolation room with negative pressure.

## Methods

Experiments were performed by measuring the exhaled air distances and directions during coughing in a high-fidelity HPS (Medical Education Technologies Inc, Sarasota, FL) in a hospital isolation room setting so as to reflect actual hospital environment. The dimension of the isolation room was 4.1×5.1×2.6 m and the room ventilation system was set at a pressure of −7.4 Pa and 16 air changes per hour.

### High-fidelity Human Patient Simulator

The HPS represented a 70-kg adult male sitting on a 45°-inclined hospital bed. The HPS contains a realistic airway and a lung model that has been applied in previous studies to simulate human respiration [20], [21]. Specifically, the simulator removed oxygen and injected carbon dioxide into the system according to a preset respiratory exchange ratio and O_2_ consumption. Coughing bouts were generated by adjusting the inspiratory pressure, so that a short burst (0.2 s duration) of oxygen flow at 650 L/min was produced for a normal coughing effort [22]. Flow rates were reduced to 320L/min and 220 L/min to simulate coughing efforts in moderate and severe lung injury, leading to mild and poor coughs, respectively [23].

### Flow Visualization

Visualization of airflow around the HPS was facilitated by marking air with smoke particles produced by a M-6000 smoke generator (N19, DS Electronics, Sydney, Australia) as described in our previous studies[13]–[19]. The oil-based smoke particles, measuring less than 1 µm in diameter, are known to follow the airflow pattern with negligible slip [16]. The smoke was introduced to the right main bronchus of the HPS. It mixed with alveolar gas, and then exhaled through the airway. Sections through the leakage jet plume were then revealed by a thin laser light-sheet (Green, 532 nm wavelength) generated by a Diode-Pumped Solid State (DPSS) laser (OEM UGH-800 mW, Lambdapro Technologies, China), with custom cylindrical optics[13]–[19].

**Figure 1:**
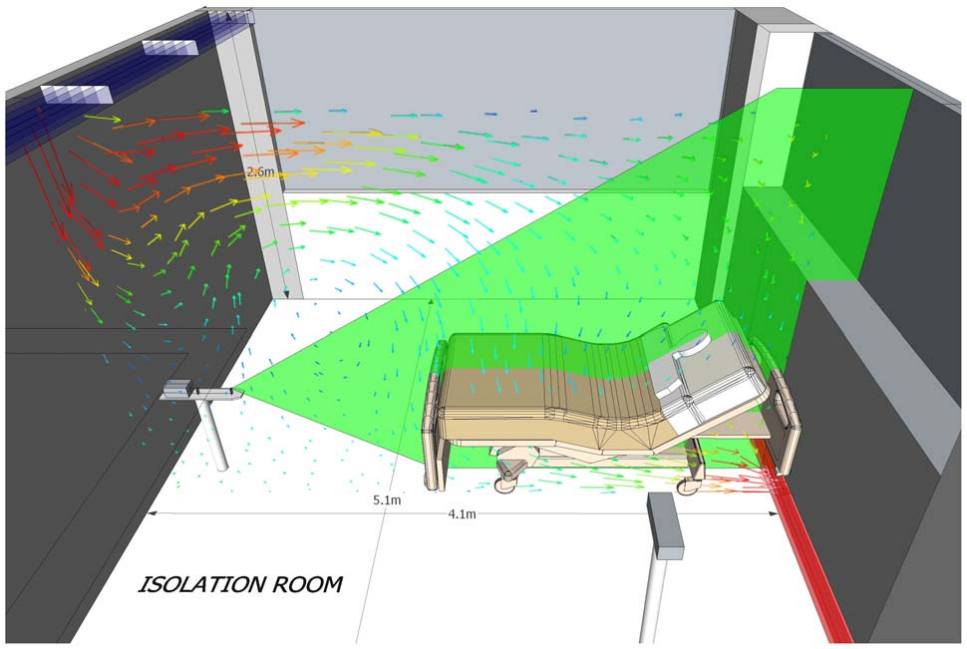
Room ventilation design and experimental set-up. The isolation room is fitted with the downward ventilation systems. The design is to supply conditioned and clean air from the ceiling diffuser to sweep away contaminants, which would then be removed via the exhaust outlets at the floor level. The Human Patient Simulator was lying at 45° on the bed. The exhaled air plume was marked with intrapulmonary smoke, and was revealed by the laser light sheet. The images were captured by a high-definition camera positioned to the left side of the simulator when examining the cough propagation distances in the median sagittal plane. Smoke concentration in the plume was estimated from the light scattered by smoke particles.

The laser light-sheet was initially positioned in the median sagittal plane of the HPS and was subsequently shifted to the paramedian planes. This allowed us to investigate the regions directly above and lateral to the mask of the HPS[13]–[19]. All coughing jet plume images, with and without wearing a surgical mask [Safe+Mask, AR Medicom Inc(Asia) Ltd, HK] or a N95 mask (M1860, 3 M, MN, USA), revealed by the laser light-sheet were captured by the high definition video camera (Sony High-Definition digital video camcorder, HDR-SR8E ClearVid complementary metal oxide semiconductor Sensor, Carl Zeiss^®^ Vario-Sonnar T* Lens, Jena, Germany), with optical resolution of 1,440×1,080 pixels per video frame (Figure 1). We exchanged the positions of the laser device and the camera when examining the lateral dispersion distances of coughs.

### Image Analysis

We estimated normalized smoke concentration in the exhaled air from the light scattered by the particles. The analysis was based on principle that the intensity of scattered light was proportional to particle concentration under the conditions that the intensity of laser illumination and the size and shape of the smoke particles were constant (i.e. monodispersity) [24].

### Image Capture and Frame Extraction

The motion video of 20 coughing episodes was captured and individual frames extracted as gray scale bitmaps for intensity analysis. Frames were extracted from the beginning of each cough, to generate an ensemble average for the corresponding instant of the coughing bouts. The time at which the normalized concentration contours spread over the widest region, from the mouth of the HPS, was chosen for the ensemble average to estimate the greatest dispersion distance[13]–[19].

### Intensity Averaging and Concentration Normalization

All gray scale frames were read into a program specifically developed for this study (MathCad 8.0, Cambridge, MA, USA) along with background intensity images taken with the laser switched off [25] The background intensity image was subtracted from each frame, pixel by pixel to remove any stray background light and the pixel intensity values were averaged over all frames to determine the ensemble averaged intensity. The resulting image was the total intensity of light scattered perpendicular to the light sheet by the smoke particles and was directly proportional to smoke concentration under the conditions mentioned above. The image was normalized against the highest intensity found within the leakage jet plume to generate normalized particle concentration contours[13]–[19].

As the smoke particles marked air that was originated from the airways of the HPS before leaking from the mask, the concentration contours effectively represented the probability of encountering air around the patient that had come from within the mask and/or the patient’s respiratory system. The normalized concentration contours were made up of data collected from 20 coughs. A contour value of 1 indicated a region that consisted entirely of air exhaled by the patient, where there was a very high chance of exposure to the exhaled air. A value near 0 indicated no measurable air leakage in the region and a small chance of exposure to the exhaled air. Significant exposure was arbitrarily defined as where there was at least 20% of normalized smoke concentration[13]–[19].

As this study did not involve human subjects, patient consent and the approval of Internal Review Boards were not required. The study received approval for non-ionizing radiation and biological/chemical safety by the Chinese University of Hong Kong.

### Statistics

The dispersion distance was expressed as mean ± standard deviation (SD). A generalized linear model was used to estimate the difference in exhaled air dispersion among different masks and during varying extent of lung injury. A 2-tailed *p* value < 0.05 was considered as statistically significant.

## Results

**Figure 2:**
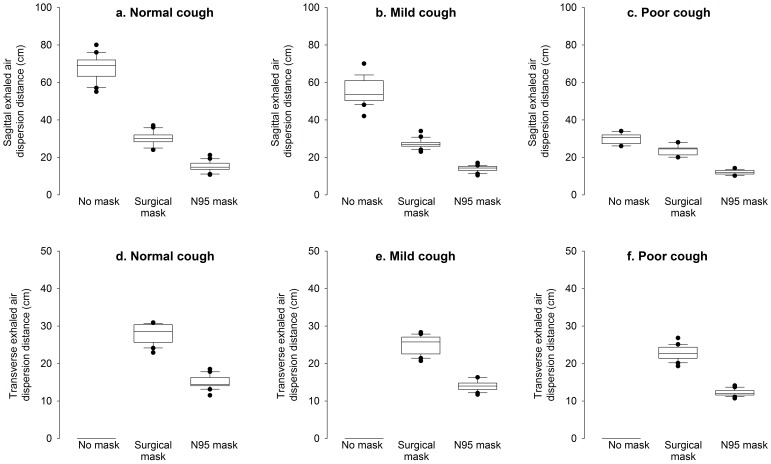
Changes of cough propagation distances in the sagittal (a–c) and transverse plane (d–f) in normal lung condition, moderate and severe lung injury when the human patient simulator was wearing no mask, a surgical mask and a N95 mask respectively. Box and whiskers plot: the upper and lower edges of the boxes indicate the interquartile ranges, the line through box is the median value, the whiskers are the 5% and 95% centiles and the closed circles are outliers.

**Figure 3:**
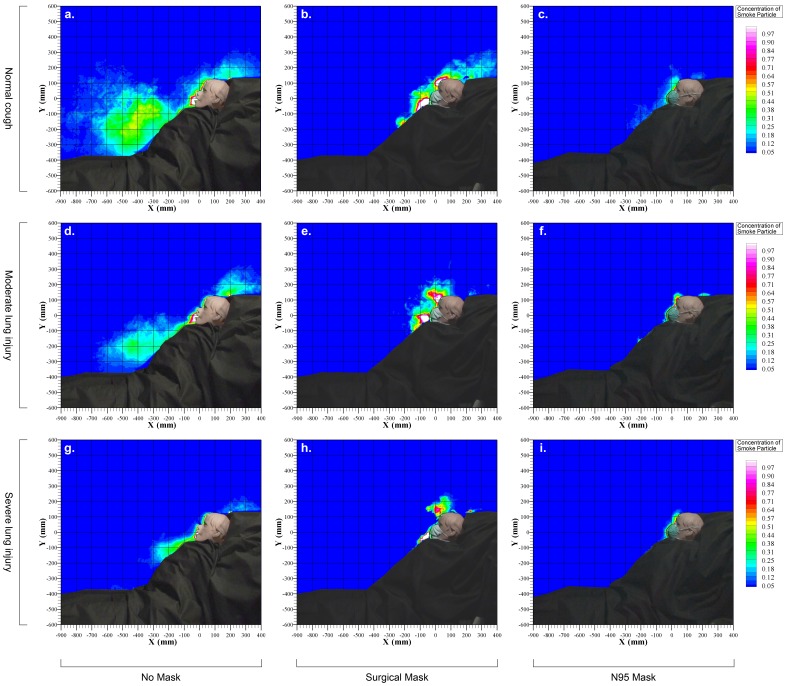
Exhaled air dispersion distances along the median sagittal plane during coughing with and without wearing a surgical or N95 mask. Normalized concentration in the plume was estimated from the light scattered by smoke particles by computer analysis. The white color code and the red color code represented regions consisting of 100% and 70% respectively of exhaled air whereas the background of the isolation room (deep blue code) was essentially free of exhaled air.

**Figure 4:**
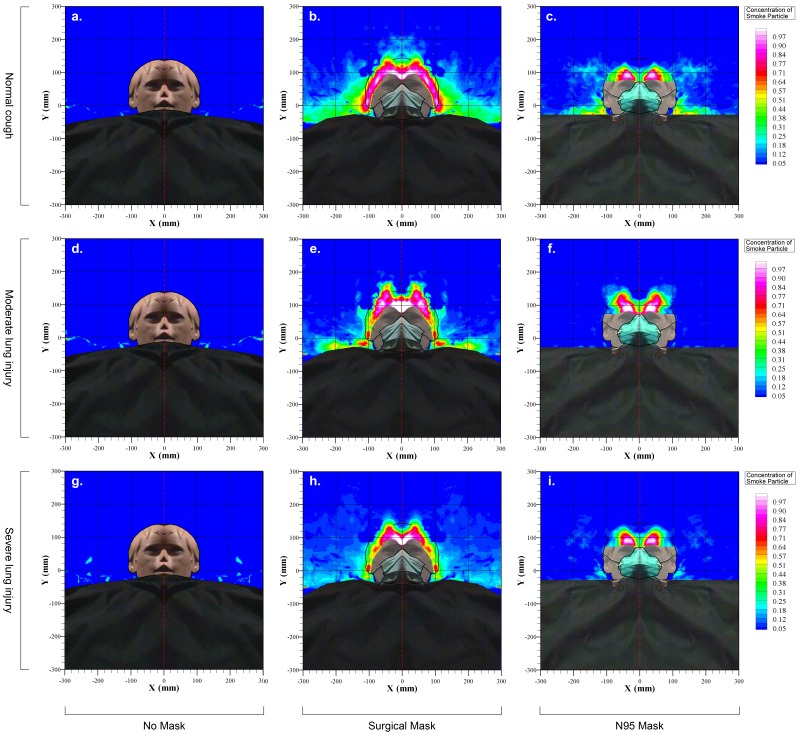
Exhaled air dispersion distances along the transverse plane during coughing with and without wearing a surgical or N95 mask.

The peak and mean cough velocities during normal, moderate and weak coughing efforts were estimated by analyzing data from 12 coughing cycles in each mode (Table 1). During normal coughing efforts when the HPS was lying at 45° on the bed, the average exhaled air dispersion distance along the median sagittal plane was 68.0 ± 6.5 cm without a facemask. This was significantly reduced by wearing a surgical mask (30.0 ± 3.4 cm) or N95 mask (15.1 ± 2.7 cm), *p* < 0.001 (Fig. 2). Exhaled air dispersion, while coughing without wearing any facemask, was in the forward and downward directions without any lateral dispersion (Fig. 3 and Fig. 4). There was substantial leakage through the mask-nasal bridge interface to the upward direction and some downward leakage through the lower edges when wearing a surgical mask (Fig. 3). With tight application of the N95 mask, there was less expelled air leakage through the nasal bridge to the upward direction (Fig. 4). The corresponding lateral dispersion distance when wearing a surgical mask was 27.9 ± 2.6 cm whereas N95 mask significantly reduced the lateral dispersion to 15.0 ± 1.7 cm, *p* < 0.001 (Fig. 4).

**Table 1:** Peak and mean cough velocities during different coughing efforts estimated by 12 cough cycles.

Coughing efforts	Peak cough velocity	95%CI	Mean cough velocity	95%CI
Normal	7.39±0.25	7.23–7.55	3.36±0.40	3.10–3.61
Moderate	6.72±0.23	6.57–6.86	2.74±0.26	2.57–2.90
Weak	5.45±0.29	5.26–5.63	1.49±0.32	1.29–1.69

* Peak and mean cough velocities are expressed in m/s.

The sagittal expelled air dispersion distances were significantly decreased in moderate (55.0 ± 6.7 cm) and severe lung injury (30.0 ± 2.6 cm, *p* < 0.001). The use of surgical mask or N95 mask further reduced the dispersion distance to 27.2 ± 2.4 cm, 14.0 ± 1.6 cm (*p* < 0.001) in moderate lung injury and 24.0 ± 2.4 cm, 12.1 ± 1.2 cm in severe lung injury, respectively. The exhaled air dispersion in moderate and severe lung injury without wearing any mask was again in the forward and downward direction, but there was again substantial leakage through the mask-nasal bridge interface to the upward direction and some downward leakage when wearing a surgical mask. The N95 mask prevented the forward leakage well, with slight leakage through the nasal bridge to the upward direction (Fig. 3 and Fig. 4).

## Discussion

As there is no reliable and safe marker that can be introduced into human lungs for study of cough aerodynamics, we have adopted the established laser smoke visualization method and the HPS model for this study[13]–[19]. We have demonstrated that coughing bouts produced a turbulent jet close to 0.7 m towards the end of the bed from the recumbent high fidelity HPS lying at 45° in a hospital isolation room with negative pressure. During normal cough, exhaled air dispersion distances along the median sagittal plane was significantly reduced to ≤ 30 cm when the HPS wore either a surgical mask or a N95 mask. With worsening lung injury, the forward dispersion distances were further reduced, so that the median sagittal distnace was ≤ 12.1 cm with the N95 mask. Lateral dispersion distances during normal cough were 28 cm and 15 cm when the HPS wore a surgical mask and a N95 mask, respectively. Thus N95 mask was more effective than surgical mask in preventing air leakage forward during coughing but there was still substantial sideway leakage.

During the influenza A (H1N1) 2009 pandemic, hospital-acquired infection had occurred in healthcare workers (HCWs) [26] and patients [27]. Front-line HCWs had higher sero-prevalence than other hospital staff or the general population in a study in Taiwan presumably due to greater exposure, [28] whereas several nosocomial outbreaks occurred in hospitalized immuno-compromised patients[29]–[31]. Infection control measures recommended by the WHO during the influenza pandemic included droplet and standard precautions among the HCWs in addition to maintaining a minimum distance of > 1 m between patients when providing routine care to patients infected with pandemic H1N1 influenza and those with influenza-like symptoms [12]. Our coughing propagation distances from the HPS would support the WHO recommendation for the HCW to wear a medical mask if working within approximately 1 m from the patient or upon entering the room/cubicle of a patient on droplet precautions [12]. Surgical masks appeared to be as effective as N95 respirators for respiratory protection of HCWs during the routine care of patients hospitalized with seasonal influenza, [32], [33] and were also as effective in reducing production of infectious aerosols during coughing when worn by influenza patients [34]. The incidence of nosocomial pandemic H1N1 infections appeared to remain low in staff members using surgical masks in a hospital despite admitting substantial numbers of pandemic H1N1 patients [35]. A systematic review has shown that the surgical masks perform similar to the N95 masks in reducing the transmission of respiratory viruses but the latter are more expensive, uncomfortable and irritating to the skin [36]. Recently, Noti et al have shown that about two-thirds of influenza virus particles from a coughing manikin (in a simulated patient room with a high-efficiency particulate air filter) were blocked from entering the mouth by surgical masks or N95 masks that were not properly fitted whereas over 99% of infectious virus particles were blocked by a well-fitted N95 mask on the breathing simulator [37]. Nevertheless it seems more practical for the HCWs to wear the surgical masks as a preventive measure against nosocomial infections in view of its reasonable filtering capacity, better comfort, and higher compliance.

Patients with influenza or other highly infectious conditions such as TB are often required to put on a facemask during acute hospital admission in order to minimize the risk of causing nosocomial infection. In a laboratory room setting, Tang et al[38]–[40] examined cough aerodynamics in healthy subjects in the upright position using the Schlieren and shadowgraph imaging techniques. It was estimated that voluntary human coughing led to airflow propagation distances of 0.55 m and 0.64 m in females and males respectively, [40] but wearing a N95 mask could block the formation of the jet whereas a surgical mask could redirect it in a less harmful direction around the top, bottom and the sides of the mask [39]. The shadowgraph imaging technique was unable to visualize the cough airflows once the temperature cooled to that of the ambient air and might have under-estimated the full cough propagation distance [40]. Our current study was able to estimate the normalized concentrations of expelled coughing particles at different distances from the recumbent patient with different extent of lung injury, in addition to showing similar distribution of the coughing plume with and without coverage by the surgical or N95 mask to Tang et al [39], [40]. Although the N95 mask was relatively more effective than the surgical mask in reducing air leakage forward from the patient during normal coughing (from 68 cm without wearing any mask to 15 cm and 30 cm), our study has revealed important novel findings that there was still substantial sideway leakage to 15 cm and 28 cm despite wearing the two different masks respectively.

Our study was limited by the use of smoke particles as markers for exhaled air. Evaporation of water content in some respiratory droplets produced by coughing would produce droplet nuclei suspended in air, whereas the larger droplets would fall to the ground in a trajectory pathway. As the smoke particles in this study mark the continuous air phase, our results would therefore represent the “upper bound” estimates for the dispersion of droplets which would be expected to follow a shorter trajectory than the air jet due to gravitational effects, but not fully reflect the risk of large droplet transmission[13]–[19]. Although the coughing efforts in this study were artificial, the peak cough velocity during normal coughing was close to the healthy human peak coughing data of 8 m/s observed by Tang et al with the Schlieren optical method^38^ and within the maximum derived velocities of 3.2–14 m/s for males measured by Shadowgraph imaging [40]. Pantelic et al [41] have demostrated the initial puff velocity produced by a cough machine as about 10 m/s whereas other investigators have reported human peak cough velocities measured by particle image velocimetry as 6 to 22 m/s [42], [43]. The variations are likely related to different areas of mouth opening [44] and the gender of the subjects [40], [43].

In summary, this study has demonstrated that coughing bouts produced a turbulent jet close to 0.7 m towards the end of the bed from the recumbent patient lying at 45° in a hospital isolation room with negative pressure. N95 mask was relatively more effective than surgical mask in preventing air leakage forward during coughing but there was still substantial sideway leakage to 15 cm. HCWs should take appropriate infection control precaution when examining or nursing hospitalized patients with potential airborne infectious diseases or severe pneumonia of unknown aetiology at an antero-lateral radial distance of 30 cm even if the patients are wearing surgical or N95 masks.

## References

[1] Musher DM (2003) How contagious are common respiratory tract infections? N Engl J Med 348: 1256–1266. 12660390 10.1056/NEJMra021771

[2] Tang JW, Li Y, Eames I, Chan PK, Ridgway GL (2006) Factors involved in the aerosol transmission of infection and control of ventilation in healthcare premises. J Hosp Infect 64: 100–114. 16916564 10.1016/j.jhin.2006.05.022 PMC7114857

[3] Tang JW, Li Y (2007) Transmission of influenza A in human beings. Lancet Infect Dis 7: 758. 18045554 10.1016/S1473-3099(07)70268-2 PMC7129893

[4] Yang W, Elankumaran S, Marr LC (2011) Concentrations and size distributions of airborne influenza A viruses measured indoors at a health centre, a day-care centre and on aeroplanes. J R Soc Interface 8: 1176–1184. 21300628 10.1098/rsif.2010.0686 PMC3119883

[5] Lindsley WG, Blachere FM, Thewlis RE, Vishnu A, Davis KA, et al (2010) Measurements of airborne influenza virus in aerosol particles from human coughs. PLoS One 5: e15100. 21152051 10.1371/journal.pone.0015100 PMC2994911

[6] Fabian P, McDevitt JJ, DeHaan WH, Fung RO, Cowling BJ, et al (2008) Influenza virus in human exhaled breath: an observational study. PLoS One. 3(7): e2691. 10.1371/journal.pone.0002691 PMC2442192 18628983

[7] Wong BC, Lee N, Li Y, Chan PK, Qiu H, et al (2010) Possible role of aerosol transmission in a hospital outbreak of influenza. Clin Infect Dis 51: 1176–1183. 20942655 10.1086/656743 PMC7107804

[8] Hui DS (2008) Review of clinical symptoms and spectrum in humans with influenza A/H5N1 infection. Respirology (Suppl. 1): S10–13. 10.1111/j.1440-1843.2008.01247.x 18366521

[9] Hui DS, Lee N, Chan PK (2010) Clinical Management of Pandemic (H1N1) Infection. Chest 137: 916–925. 20022969 10.1378/chest.09-2344 PMC7094244

[10] Lee N, Hui DS, Wu A, Chan P, Cameron P, et al (2003) A major outbreak of severe acute respiratory syndrome in Hong Kong. N Engl J Med 348: 1986–1994. 12682352 10.1056/NEJMoa030685

[11] Tang JW, Settles GS (2008) Images in clinical medicine. Coughing and aerosols. N Engl J Med 359(15): e19. 18843121 10.1056/NEJMicm072576

[12] WHO (2009) Infection prevention and control during healthcare for confirmed, probable or suspected cases of pandemic (H1N1) 2009 virus infection and influenza like illness -updated guidance. Available: http://www.who.int/csr/resources/publications/cp150_2009_1612_ipc_interim_guidance_h1n1.pdf. Accessed 2012 Jan 21.

[13] Hui DS, Ip M, Tang JW, Wong AL, Chan MT, et al (2006) Airflows around oxygen masks: A potential source of infection? Chest 130: 822–826. 16963681 10.1378/chest.130.3.822 PMC7094573

[14] Hui DS, Hall SD, Chan MT, Chow BK, Tsou JY, et al (2006) Non-invasive positive pressure ventilation: An experimental model to assess air and particle dispersion. Chest 130: 730–740. 16963670 10.1378/chest.130.3.730 PMC7094473

[15] Hui DS, Hall SD, Chan MT, Chow BK, Ng SS, et al (2007) Exhaled air dispersion during oxygen delivery via a simple oxygen mask. Chest 132: 540–546. 17573505 10.1378/chest.07-0636 PMC7094533

[16] Hui DS, Chow BK, Hall SD, Ng SS, Hall SD, et al (2009) Exhaled air and aerosolized droplet dispersion during application of a jet nebulizer. Chest 135: 648–654. 19265085 10.1378/chest.08-1998 PMC7094435

[17] Hui DS, Chow BK, Hall SD, Chu LC, Hall SD, et al (2009) Exhaled air dispersion distances during application of non-invasive ventilation via different Respironic face masks. Chest 136: 998–1005. 19411297 10.1378/chest.09-0434 PMC7094372

[18] Hui DS, Chow BK, Chu L, Ng SS, Lai ST, et al (2011) Exhaled air dispersion and removal is influenced by isolation room size and ventilation settings during oxygen delivery via nasal cannula. Respirology 16: 1005–1013. 21605275 10.1111/j.1440-1843.2011.01995.x

[19] Chan MTV, Chow B, Chu Leo, Hui DS (2012) Mask ventilation and dispersion of exhaled air. Am J Respir Crit Care Med (in press). 10.1164/rccm.201201-0137im 23540885

[20] Goodwin JA, van Meurs WL, Sa Couto CD, Beneken JE, Graves SA (2004) A model for educational simulation of infant cardiovascular physiology. Anesth Analg 99: 1655–1664. 15562049 10.1213/01.ANE.0000134797.52793.AF

[21] Lampotang S, Lizdas DE, Gravenstein N, Robicsek S (2006) An audible indication of exhalation increases delivered tidal volume during bag valve mask ventilation of a patient simulator. Anesth Analg 102: 168–171. 16368824 10.1213/01.ANE.0000181833.23904.4E

[22] Lavietes MH, Smeltzer SC, Cook SD, Modak RM, Smaldone GC (1998) Airway dynamics, oesophageal pressure and cough. Eur Respir J 11: 156–161. 9543286 10.1183/09031936.98.11010156

[23] Suárez AA, Pessolano FA, Monteiro SG, Ferreyra G, Capria ME, et al (2002) Peak flow and peak cough flow in the evaluation of expiratory muscle weakness and bulbar impairment in patients with neuromuscular disease. Am J Phys Med Rehabil 81: 506–511. 12131177 10.1097/00002060-200207000-00007

[24] Soo SL (1967) Fluid dynamics of multiphase systems. Toronto: Blaisdell Publishing Company.

[25] MathSoft Inc (2000) Mathcad 8.0 for windows, users guide. Cambridge, MA, USA, MathSoft Inc.

[26] Wise ME, De Perio M, Halpin J, Jhung M, Magill S, et al.. (2011) Transmission of pandemic (H1N1) 2009 influenza to healthcare personnel in the United States. Clin Infect Dis (Suppl 1): S198–204. 10.1093/cid/ciq038 21342895

[27] Enstone JE, Myles PR, Openshaw PJ, Gadd EM, Lim WS, et al (2011) Nosocomial pandemic (H1N1) 2009, United Kingdom, 2009–2010. Emerg Infect Dis 17: 592–598. 21470446 10.3201/eid1704.101679 PMC3377421

[28] Chan YJ, Lee CL, Hwang SJ, Fung CP, Wang FD, et al (2010) Seroprevalence of Antibodies to Pandemic (H1N1) 2009 Influenza Virus Among Hospital Staff in a Medical Center in Taiwan. J Chin Med Assoc 73: 62–66. 20171584 10.1016/S1726-4901(10)70003-4 PMC7129009

[29] Moore C, Galiano M, Lackenby A, Abdelrahman T, Barnes R, et al (2011) Evidence of person-to-person transmission of oseltamivir-resistant pandemic influenza A(H1N1) 2009 virus in a hematology unit. J Infect Dis 203: 18–24. 21148492 10.1093/infdis/jiq007 PMC3086444

[30] Buchbinder N, Dumesnil C, Pinquier D, Merle V, Filhon B, et al (2011) Pandemic A/H1N1/2009 influenza in a paediatric haematology and oncology unit: successful management of a sudden outbreak. J Hosp Infect 79: 155–160. 21783276 10.1016/j.jhin.2011.04.019

[31] Chen LF, Dailey NJ, Rao AK, Fleischauer AT, Greenwald I, et al (2011) Cluster of oseltamivir-resistant 2009 pandemic influenza A (H1N1) virus infections on a hospital ward among immunocompromised patients–North Carolina, 2009. J Infect Dis 203: 838–846. 21343149 10.1093/infdis/jiq124 PMC3307091

[32] Jefferson T, Del Mar C, Dooley L, Ferroni E, Al-Ansary LA, et al (2009) Physical interventions to interrupt or reduce the spread of respiratory viruses: systematic review. BMJ 339: b3675. 19773323 10.1136/bmj.b3675 PMC2749164

[33] Loeb M, Dafoe N, Mahony J, John M, Sarabia A, et al (2009) Surgical mask vs N95 respirator for preventing influenza among health care workers: a randomized trial. JAMA 302: 1865–1871. 19797474 10.1001/jama.2009.1466

[34] Johnson DF, Druce JD, Birch C, Grayson ML (2009) A quantitative assessment of the efficacy of surgical and N95 masks to filter influenza virus in patients with acute influenza infection. Clin Infect Dis 49: 275–277. 19522650 10.1086/600041

[35] Ang B, Poh BF, Win MK, Chow A (2010) Surgical Masks for Protection of Health Care Personnel against Pandemic Novel Swine-Origin Influenza A (H1N1)-2009: Results from an Observational Study. Clin Infect Dis 50: 1011–1014. 20178418 10.1086/651159

[36] Jefferson T, Del Mar CB, Dooley L, Ferroni E, Al-Ansary LA, et al (2011) Physical interventions to interrupt or reduce the spread of respiratory viruses. Cochrane Database Syst Rev (7): CD006207. 10.1002/14651858.CD006207.pub4 PMC6993921 21735402

[37] Noti JD, Lindsley WG, Blachere FM, Cao G, Kashon ML, et al (2012) Detection of infectious influenza virus in cough aerosols generated in a simulated patient examination room. Clin Infect Dis 54: 1569–1577. 22460981 10.1093/cid/cis237 PMC4680957

[38] Tang JW, Settles GS (2009) Images in clinical medicine. Coughing and masks. N Engl J Med 361(26): e62. 20032322 10.1056/NEJMicm0904279

[39] Tang JW, Liebner TJ, Craven BA, Settles GS (2009) A schlieren optical study of the human cough with and without wearing masks for aerosol infection control. J R Soc Interface 6 (Suppl 6)S727–736. 19815575 10.1098/rsif.2009.0295.focus PMC2843945

[40] Tang JW, Nicolle A, Pantelic J, Koh GC, Wang LD, et al (2012) Airflow dynamics of coughing in healthy human volunteers by shadowgraph imaging: an aid to aerosol infection control. PLoS One 7: e34818. 22536332 10.1371/journal.pone.0034818 PMC3335026

[41] Pantelic J, Sze-To GN, Tham KW, Chao CY, Khoo YC (2009) Personalized ventilation as a control measure for airborne transmissible disease spread. J R Soc Interface 6 Suppl 6S715–726. 19812074 10.1098/rsif.2009.0311.focus PMC2843944

[42] Zhu S, Kato S, Yang JH (2006) Study on transport characteristics of saliva droplets pruduced by coughing in a calm indoor environment. Build Environ 41: 1691–1702.

[43] Kwon SB, Park J, Jang J, Cho Y, Park DS, et al (2012) Study on the initial velocity distribution of exhaled air from coughing and speaking. Chemosphere 87(11): 1260–1264. 22342283 10.1016/j.chemosphere.2012.01.032 PMC7112028

[44] Gupta JK, Lin CH, Chen Q (2009) Flow dynamics and characterization of a cough. Indoor Air 19: 517–525. 19840145 10.1111/j.1600-0668.2009.00619.x

